# A new species of *Boloponera* from Sekhukhuneland, South Africa (Hymenoptera, Formicidae, Ponerinae)

**DOI:** 10.3897/zookeys.798.28606

**Published:** 2018-11-21

**Authors:** Peter G. Hawkes

**Affiliations:** 1 AfriBugs CC, 341 27th Avenue, Villieria, Pretoria, Gauteng Province, 0186, South Africa AfriBugs Pretoria South Africa; 2 Department of Zoology, University of Venda, Thohoyandou, Limpopo Province, South Africa University of Venda Thohoyandou South Africa

**Keywords:** Afrotropical, *
Boloponera
*, conservation, range extension, taxonomy

## Abstract

During an environmental impact assessment survey of a proposed tailings storage facility for a platinum mine in Sekhukhuneland, South Africa, five adult and five larval specimens of a new species of *Boloponera* were found while excavating soil to a depth of 10–15 cm at the base of a tree in riparian woodland. These specimens represent a 3400 km range extension and the first reported record of the genus since its description in 2006, which was based on a single specimen collected in the Central African Republic in 2001. A description of the worker and ergatoid queen of *Boloponeraikemkha***sp. n**. is presented, with a description of the mature larva and a key to distinguish workers of the two currently known species of the genus. The taxonomic relationships of *Boloponera* are discussed with respect to several confirmed and newly identified autapomorphies that support its retention as a distinct genus, although closely related to *Plectroctena* and *Loboponera*. A preliminary assessment of the conservation status and discussion of potential threats to the survival of *B.ikemkha* is also provided. Evaluation of current data under the IUCN Red List criteria would result in *B.ikemkha* being assessed as Critically Endangered, but further investigation is required to test the validity of placing it in this category.

## Introduction

*Boloponera* was described by Fisher (2006) on the basis of a single worker specimen collected in the Central African Republic in 2001 and, with no further specimens apparently having been recorded since (Fisher and Bolton 2016), it is one of the most rarely encountered African ant genera known. During an environmental impact assessment (EIA) of a proposed tailings storage facility (TSF) for the Two Rivers Platinum Mine (TRPM) in Limpopo Province, South Africa, five adult specimens and five larvae of a previously unknown species of *Boloponera* were collected, representing a 3400 km range extension for the genus.

The single known specimen of the previously described *B.vicans* Fisher lacks eyes, but while three of the newly collected specimens also lack eyes, two have moderately well-developed compound eyes with *circa* 15 ommatidia each; these specimens are also larger and more stoutly built and in particular have larger gasters and relatively longer legs. While the possibility that the specimens with eyes represent a distinct major worker caste cannot entirely be excluded, it seems most likely that they are ergatoid queens. An eyeless specimen is thus designated as the holotype worker and a list of the characters distinguishing the putative ergatoid queens is presented.

A description of the new species is provided and includes a description of larval morphology, an update of some of the diagnostic characters of *Boloponera* and a key to distinguish the two currently known species of the genus. The taxonomic relationships between *Boloponera*, *Loboponera* and *Plectroctena* are discussed with respect to several confirmed and newly identified *Boloponera* autapomorphies. An assessment of the conservation status of *B.ikemkha* sp. n. and potential threats to the survival of the species is also presented.

## Materials and methods

Measurements were taken using a Leica MZ16 stereomicroscope equipped with an axial shift carrier and an ocular graticule calibrated against a stage micrometer; specimens were photographed using a Leica DFC 425 digital camera connected to the same microscope. Multifocus images were captured using Leica Application Systems (LAS); montage images were generated using Helicon Focus V6.2.0 and edited with Adobe Photoshop CS3. Scanning electron microscope (SEM) images of larvae (carbon coated using an Emitech K950 X Turbo Evaporator) were taken with a Zeiss Gemini Crossbeam 540 field emission SEM.

### Terminology

Terminology relating to adult morphology follows Fisher and Bolton (2016) and Keller (2011), descriptions of surface sculpture follow Harris (1979) and larval morphology follows Wheeler and Wheeler (1976a, b). Of particular significance are the lobes covering the antennal insertions, which were termed frontal lobes in Fisher’s (2006) description of *Boloponeravicans* and in previous treatments of members of the *Plectroctena* genus-group. Keller (2011) coined the term *torular lobe* to describe these structures, which are formed from a laterad expansion of the highest parts of the median arches of the toruli and are not homologous to the frontal lobes of other ants. Also important is the *ventral flap on metapleural gland opening*, a term used by Keller (2011) for a thin cuticular extension of the ventral margin of the orifice of the metapleural gland.

### Measurements

**Gt1L** Maximum length of first gastral tergite (A3) in dorsal view

**Gt1W** Maximum width of first gastral tergite (A3) in dorsal view

**Gt2L** Maximum length of second gastral tergite (A4) in dorsal view

**Gt2W** Maximum width of second gastral tergite (A4) in dorsal view

**HH** Head height: maximum height of head in profile view, measured perpendicular to the longitudinal axis of the head capsule

**HL** Head Length: length of head measured in full-face view, from the midpoint of the clypeal margin to the midpoint of the posterior margin; where either of these margins is concave the measurement is taken from the midpoint of a line joining the anterior-most portions of the clypeus or the posterior-most portions of the posterior margin

**HW** Head Width: maximum width of head, measured in full-face view

**ML** Maximum length of mandible from apex to intersection with the outer clypeal margin

**MesTL** Maximum length of dorsal surface of the mesotibia measured from the proximal constriction to the apex

**MetTL** Maximum length of dorsal surface of the metatibia measured from the proximal constriction to the apex

**OD** Ocular Diameter: maximum diameter of the eye, measured with head in profile view

**PeNH** Maximum height of petiole node in profile view, excluding the subpetiolar process

**PeH** Maximum height of petiole in profile view, including the subpetiolar process

**PeW** Maximum width of petiole in dorsal view

**PeNL** Maximum length of petiole node in profile view

**PeL** Maximum length of petiole in profile view, including anterior and posterior articulations

**ProTL** Maximum length of dorsal surface of the protibia measured from the proximal constriction to the apex

**PW** Pronotal Width: maximum width of pronotum measured in dorsal view

**SL** Scape Length: maximum straight-line length of the scape, excluding the basal constriction

**TL** Total Length: sum of HL + WL + PeL + gaster length (sum of gastral segment 1 + combined lengths of gastral segments 2–5 measured in lateral view at the height of the 1^st^ and 2^nd^ gastral spiracles respectively); this measure excludes the mandibles and is an approximation due mainly to variable extension and flexion of gastral segments

**TLW** Torular lobe width: maximum width across torular lobes in full-face view

**WL** Weber’s Length: diagonal length of mesosoma in profile, from the junction of the pronotum and the cervical shield, to the posterior basal angle of the metapleuron

### Indices and estimates

**CI** Cephalic Index: (HW*100)/HL

**Gt1LI** Gastral Tergite 1 Length Index: (Gt1L*100/WL)

**Gt1WI** Gastral Tergite 1 Width Index: (Gt1W*100/WL)

**Gt2LI** Gastral Tergite 2 Length Index: (Gt2L*100/WL)

**Gt2WI** Gastral Tergite 2 Width Index: (Gt2W*100/WL)

**HVe** Head Volume estimate: (HL*HW*HH)*(4π/3); while not an exact measure of cephalic volume, this estimate is directly proportional to actual volume and for a given head shape the proportionality remains constant and so allows comparison of relative cephalic volumes between individuals with similar head shapes; estimates presented in mm^3^

**OI** Ocular Index: (OD × 100)/HW

**SI** Scape Index: (SL × 100)/HW

### Abbreviations of depositories

**AFRC** AfriBugs collection, Pretoria, South Africa

**CASC** California Academy of Sciences Collection, San Francisco, USA

**SAMC** Iziko South African Museum Collection, Cape Town, South Africa

## Taxonomy

### Diagnosis of *Boloponera* worker

As in Fisher (2006), with the following additions and modifications:

1. Glandular structure present on metafemur, visible for approximately 2/3 of the length of the femur as a thinning of the cuticle, without any impression or groove. No such structure visible on mesofemur.

2. Anterior disc of petiole with a broadly C-shaped to horseshoe-shaped strip of thicker cuticle surrounding a roughly semicircular patch of thinner cuticle.

3. Anterior margin of clypeus with a strongly convergent pair of long setae that cross at approximately their mid-length.

4. Posterior margin of clypeus projecting anterad of the anterior clypeal margin and overhanging the closed mandibles.

5. Mandible with an apical tooth plus three pre-apical teeth; the sub-apical tooth is close to the apical and is followed by a large diastema before the third tooth, which is located near the fourth (basal) tooth at the basal angle, near the mid-length of the mandible.

6. Metapleural gland orifice opening dorsally oriented, clearly visible in dorsal view but largely obscured in lateral view; posteriorly it is completely hidden by the upwardly extended ventral flap.

### Key to workers of *Boloponera*

**Table:** 

1	Torular lobes distinctly longitudinally striate; first gastral tergite with standing hairs and appressed pubescence; subpetiolar process with a strongly developed posteriorly projecting acute tooth; mesosoma dorsum, scapes and femora longitudinally striate between scattered piligerous foveolae (Central African Republic)	***vicans* Fisher, 2006**
–	Torular lobes smooth and highly polished without striations; first gastral tergite with appressed pubescence only; subpetiolar process terminating in a blunt slightly recurved posteroventral point, but without a strong posteriorly projecting acute tooth; mesosoma dorsum, scapes and femora smooth and shining between scattered piligerous foveolae (South Africa)	***ikemkha* sp. n.**

#### Boloponera ikemkha sp. n.

Taxon classificationAnimaliaHymenopteraFormicidae

http://zoobank.org/8849C864-5D24-4BD6-A772-A09F2D8E9C7F

**Figure 1.**
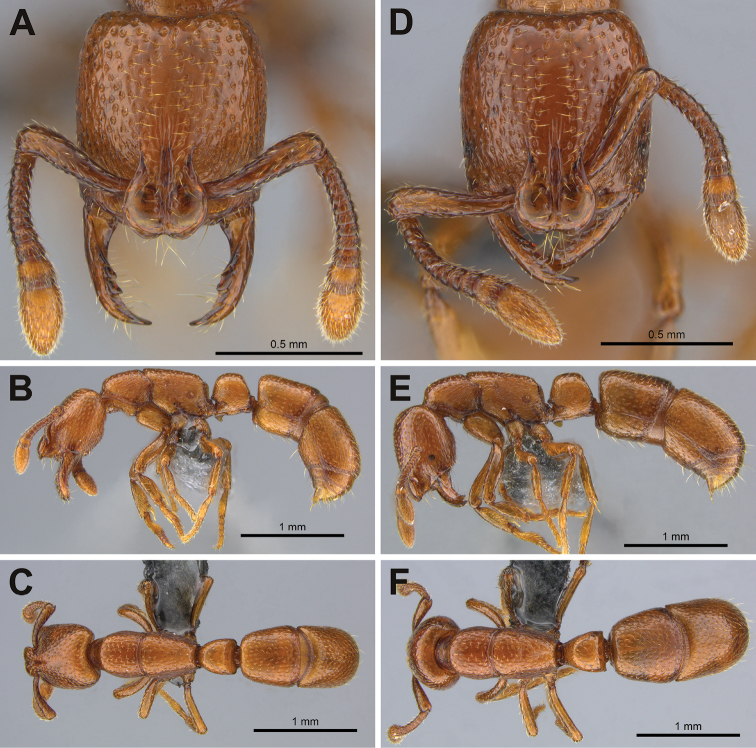
*Boloponeraikemkha*. **A–C** holotype worker, CASENT0254322**A** full-face view **B** lateral view **C** dorsal view **D–F** paratype ergatoid queen, CASENT0254320, same magnifications as worker images **D** full-face view **E** lateral view **F** dorsal view (photographs by Peter Hawkes, from www.AntWeb.org).

**Figure 2.**
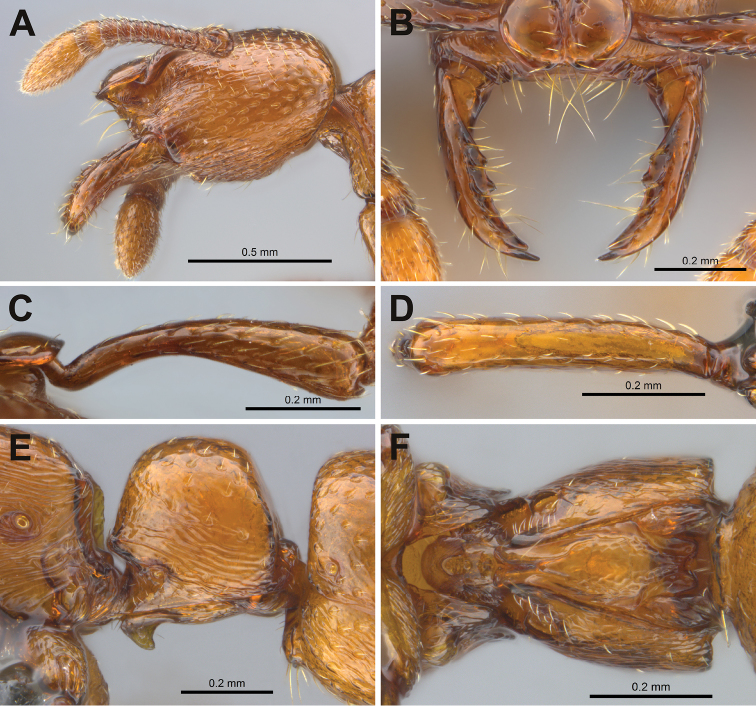
*Boloponeraikemkha*. **A, B, D, E** holotype worker CASENT0254322**C, F** paratype ergatoid queen CASENT0254320**A** head, lateral view **B** clypeus and mandibles **C** right scape, posterior view showing inflection from which length is measured **D** left metafemur, dorsal view **E** petiole, lateral view **F** petiole, ventral view (photographs by Peter Hawkes, from www.AntWeb.org).

**Figure 3.**
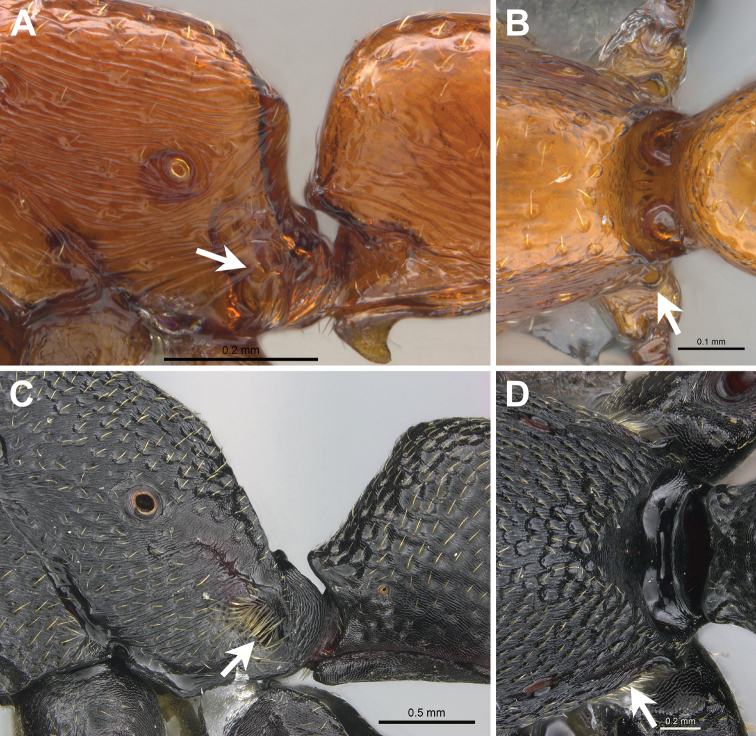
Metapleural gland openings. **A, B***Boloponeraikemkha* holotype worker, CASENT0254322**A** profile view **B** dorsal view **C, D***Plectroctenastrigosa*CASENT0235672**C** profile view **D** dorsal view. Metapleural gland openings indicated by arrows (photographs by Peter Hawkes, from www.AntWeb.org).

**Figure 4.**
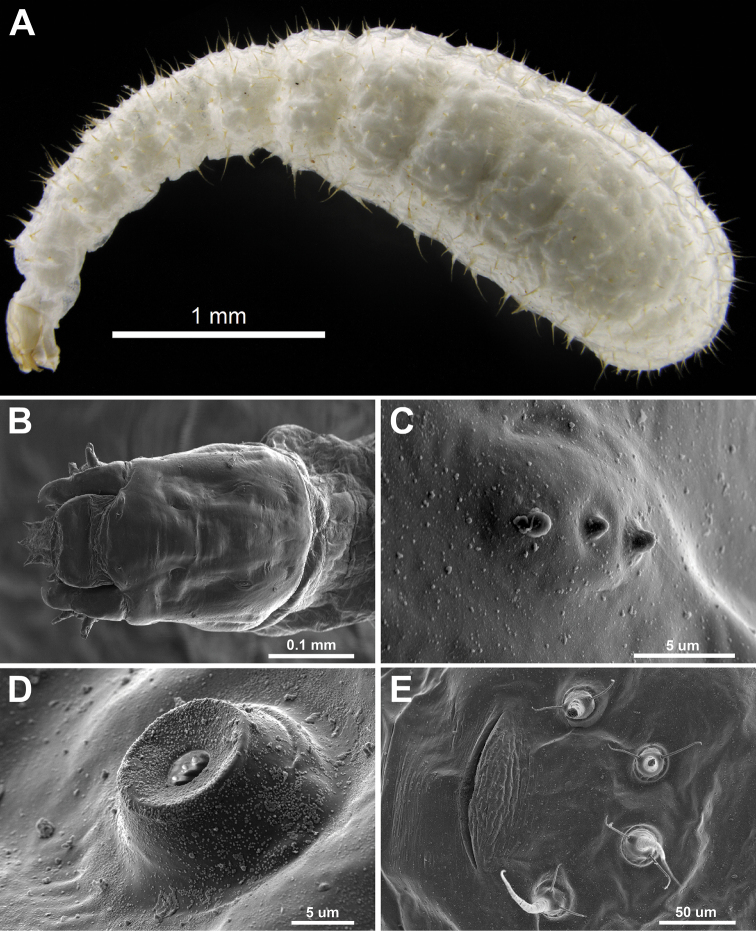
*Boloponeraikemkha* larva. **A–B**CASENT0257321**A** lateral view before coating for SEM. (Photo by Peter Hawkes, from www.AntWeb.org) **B** head, dorsal view **C–E**CASENT0257322**C** left antenna **D** left spiracle on abdominal segment 2 **E** anus with associated tubercles (SEM images by Peter Hawkes & Jonathan Fisher, from www.AntWeb.org).

Adult, Fig. 1A–F , Fig. 2A–F , Fig. 3A–B , Larva, Fig. 4A–E

##### Worker measurements

(3 measured, holotype in parentheses): TL 3.35–3.42 (3.42), HL 0.76–0.77 (0.77), HW 0.61–0.62 (0.62), HH 0.48–0.49 (0.49), SL 0.43–0.44 (0.44), TLW 0.28–0.29 (0.28), ML 0.46 (0.46), PW 0.45–0.46 (0.45), WL 1.06–1.08 (1.07), PeNH 0.38–0.39 (0.39), PeH 0.43–0.44 (0.43), PeW 0.34–0.35 (0.34), PeNL 0.36–0.37 (0.36), ProTL 0.36–0.38 (0.37), MesTL 0.30–0.32 (0.31), MetTL 0.38–0.39 (0.38), Gt1L 0.56–0.57 (0.57), Gt1W 0.57–0.58 (0.58), Gt2L 0.66–0.68 (0.68), Gt2W 0.57–0.58 (0.58). **Indices and estimates**: CI 80–82 (81), SI 70–71 (71), HVe 0.117–0.124 (0.124), Gt1WI 53–55 (55), Gt2WI 53–55 (55) (all measurements in mm except HVe, which is presented in mm^3^).

##### Description.

*Head* subrectangular, moderately longer than wide (CI 80–82), posterior margin shallowly indented medially, sides almost straight but slightly divergent in anterior half, rounding posteriorly into the broadly convex vertices. Torular lobes extremely large and protruding anteriorly over the clypeus, forcing the medial portion of the posterior margin of the clypeus anterad of, and overhanging, the medially concave anterior clypeal margin. Torular lobes translucent and highly polished, without any trace of striate sculpture except in the median strip between the lobes, a few piligerous foveolae present adjacent to this strip and in the posterior portion of the lobes. A pair of short, weakly diverging setae arise medially at the upper (= posterior) margin of the clypeus, a second similar but more strongly divergent and longer pair arise below these from about the midlength of the clypeus and a third pair of strongly convergent setae arise from the lower (= anterior) clypeal margin. The latter setae cross each other at about their midlength. Lateral portion of clypeus divided into sloping anterior and flat posterior sections by a transverse carina. Clypeus smooth and shining, weakly sculptured posterolaterally and with several weak and incomplete diagonal carinae anteriorly. Frontal carinae are very short, fading out immediately behind the torular lobes and failing to reach the mid-length of the head. Eyes and ocelli absent. Mandibles smooth and shining with scattered piligerous punctures, elongate, curved inward apically and each with an apical and a preapical tooth, with an additional blunt tooth near and another at the base of the masticatory margin. A fine but distinct groove arises dorsally at the base of the basal margin, running diagonally across the outer surface of the mandible, reaching the lateroventral margin at about one third of the length of the mandible and continuing along this margin to the apex, but no dorsal groove parallel to the masticatory margin is present. Antennal scapes short, stout, basally curved and distally thickened; when laid back, scapes fall short of the posterior margin of the head by about half their length. Antennal segment 2 slightly longer than broad, segments 3 to 10 distinctly broader than long. Two-segmented club formed by segment 11, which is slightly broader than long, and the apical (12^th^) segment, which is twice as long as broad. Scapes with strong sub-appressed pubescence only, lacking erect setae, remaining segments with appressed pubescence and short suberect setae, all segments smooth and shining, unsculptured except for piligerous punctures. Head smooth and shining with scattered piligerous foveolae everywhere apart from a small posterodorsal patch medially, the foveolae irregularly spaced but on average separated by more than their diameter. Hairs arising from the foveolae appressed and medially oriented on dorsum of head. Foveolae weakly longitudinally aligned, spaces between them smooth and shining dorsally and posteriorly but laterally, anteriorly and ventrally undose. *Mesosoma*: laterally striate, becoming undose dorsolaterally, the sculpture stronger on the pronotum and metapleura, weaker on the mesopleura. All dorsal surfaces smooth and shining medially, weakly undose laterally. Entire dorsal mesosoma with scattered piligerous foveolae which are more widely spaced medially. Promesonotal suture well-defined and flexible, metanotal groove entirely absent dorsally and only faintly discernible laterally. Katepisternum well-defined and isolated by a sharply incised suture, anepisternum also sharply defined dorsally, but not posteriorly, where it is contiguous with the metapleuron. Propodeal spiracles round, situated at about the mid-height of the sides of the propodeum. Propodeal declivity flanked by strongly developed translucent lamellae running from the posterolateral corners of the propodeum to the metapleural lobes, with which they are confluent. In profile the propodeal dorsum meets the declivity in an obtuse angle, the surfaces separated by a weakly defined arched edge that is confluent with the lateral lamellae. Declivity shallowly concave in dorsal view, mostly smooth, but weakly shagreenate in upper half. Metapleural lobes broadly rounded, incurved ventrally. Metapleural gland bulla expanded and protruding posterolaterally, the orifice opening dorsally and obscured posteriorly in lateral view by the upwardly extended ventral flap. Pretarsal claws without preapical teeth. Metafemur dorsally with a strip of thin cuticle slightly more than half its length through which an apparently glandular structure can be seen (see Figure 2D), the surface neither flattened nor indented; mesofemur lacking any such feature. *Petiole*: node in dorsal view distinctly wider behind than in front, in profile with anterior and posterior faces convergent, the node tapering to the very broadly rounded summit. Subpetiolar process keel-like, slightly recurved and produced posteriorly as a blunt point. Anterior face of node with weakly reticulate sculpture; posterior face smooth, with a few very faint short striae radiating from the posterior petiolar peduncle. Sides of node smooth posterodorsally and with undose sculpture anteroventrally restricted; dorsal surface of node smooth. Lateral and dorsal surfaces of node with scattered piligerous foveolae, which are absent from anterior and posterior faces. Anterior disc of petiole ventrally with a broad C-shaped strip of cuticle around a thinner semicircular patch (see Figure 2F). *Gaster* with tergites smooth and shining, very weakly undose between scattered piligerous foveolae, pubescence appressed to sub-appressed. Sting present, weakly curved. *Pilosity*: standing hairs absent from all dorsal surfaces of head and body apart from posterior margin of gastral tergite 2 (A4) and dorsum of subsequent tergites. Meso- and metathoracic tibiae each with a single subapical pectinate spur and a pair of elongate setae located more proximally on the ventral surface. Outer surfaces of femora as well as pro- and metathoracic tibiae with short appressed pubescence, mesothoracic tibiae with longer suberect setae. *Colour*: medium reddish-brown, legs and apical two antennal segments slightly paler.

##### Material.

**Holotype worker**. SOUTH AFRICA, Limpopo, Sekhukhune, De Grooteboom 373 KT portion 1, 1025 ±10m, -24.93625, 30.14494 ±5m, P. Hawkes, J. Fisher, S. Pillay, 08.xii.2016, TRP2016b-TSF-131, Riverine fringe forest (in Sekhukhune Mountain Bushveld), hand collected 10–15 cm deep in soil at base of tree, CASENT0254322 (SAMC).

**Paratype workers.** 2 specimens, same data as holotype, CASENT0254323 (CASC), CASENT0254324 (AFRC)

##### Ergatoid queen measurements

(2 measured): TL 3.82–3.84, HL 0.84, HW 0.70, HH 0.54–0.55, SL 0.51, OD 0.06, TLW 0.31, ML 0.48–0.49, PW 0.51, WL 1.17–1.18, PeNH 0.43, PeH 0.48, PeW 0.39–0.40, PeNL 0.40–0.41, ProTL 0.43, MesTL 0.36, MetTL 0.47, Gt1L 0.66, Gt1W 0.70, Gt2L 0.82, Gt2W 0.71–0.72. **Indices and estimates**: CI 83, SI 72–73, HVe 0.166–0.167, Gt1WI 60, Gt2WI 61, OI 9 (all measurements in mm except HVe, which is presented in mm^3^).

Matching the description of the worker but differing in the following respects:

1. Larger overall, with head relatively slightly broader and scapes relatively slightly longer (see Table 1);

2. Gastral segments 1 & 2 absolutely and relatively broader and longer (see Table 1, Figures 1B–F);

3. Mesosoma relatively very slightly broader (PW/WL 0.44 vs 0.43), metanotal suture faintly visible in dorsal view in one specimen;

4. Compound eyes present, with 12–17 rather poorly defined ommatidia of varying size and shape, making precise counts difficult;

5. Head somewhat more rounded posterolaterally (compare Figures 1A and 1D).

6. Subpetiolar process more bluntly rounded apically, not posteriorly recurved (compare Figures 1B and E).

7. Legs longer, the difference being more pronounced in the middle and hind legs (compare Figures 1C and 1F and see Table 1); in absolute terms the pro-, meso- and metathoracic tibiae of the ergatoid specimens are 14%, 17% and 21% longer respectively than those of the workers and relative to Weber’s length are 4%, 7% and 10% longer.

##### Paratype ergatoid queens.

2 specimens, same data as holotype worker, CASENT0254320 (AFRC), CASENT0254321 (CASC).

##### Male.

Unknown.

##### Etymology.

*ikemkha* is derived from Ancient Egyptian (*ikem* = shield; *kha* = shining) and refers to the very large, highly polished torular lobes. The specific epithet is a noun in apposition and is thus invariant.

**Table 1.** Comparison of worker and ergatoid queen measurements (in mm) and indices of *B.ikhemka*; values presented as mean ± standard deviation.

Caste	TL	CI	SI	Gt1WI	Gt1LI	Gt2WI	Gt2LI	ProTL/WL	MesTL/WL	MetTL/WL
Worker	**3.39** ± 0.04	**81** ± 0.70	**70** ± 0.65	**54** ± 0.64	**54** ± 0.75	**53** ± 0.73	**63** ± 0.53	**0.35** ± 0.006	**0.29** ± 0.004	**0.36** ± 0.003
Ergatoid queen	**3.83** ± 0.01	**83** ± 0.30	**73** ± 0.20	**60** ± 0.02	**61** ± 0.02	**56** ± 0.27	**70** ± 0.43	**0.36** ± 0.001	**0.31** ± 0.001	**0.40** ± 0.002

##### Larval morphology.

Mature (assumed, based on size) larva: white, length through spiracles 4.1 ± 0.7 mm (three measured), elongate pogonomyrmecoid form, weakly curved but distinctly differentiated into head, neck (T1–3 + AI–II) and body (AIII–X). Tubercles yellowish-white, very numerous (708 on CASENT0257322), conoid with 0–2 simple hairs (0–2 on T1–T3, 0–1 on AI–AX) and surmounted by an elongate slender cone with spinulose integument (= conoid with spine *sensu*Wheeler and Wheeler 1976a). Tubercles absent ventrally on T1–T3.

*Anus* ventral, a weakly recurved transverse slit approximately 0.1 mm across, with a very fine anterior and much larger posterior lip (Figure 4E), four tubercles (conoid with a spine and two setae) arranged in a semicircle just behind the anus. Spiracles visible on T2–T3 and A1–A8, all ten of similar diameter (3.5–4.0μm); each set in a short cone-like peg set in a slight depression and with spinules on the inner surface of the atrium (Figure 4D). Thoracic segments and first eight abdominal segments distinct; abdominal segments nine and ten difficult to distinguish. Spinules abundant, in scattered short transverse rows of 2–7 posteriorly, longer rows ventrally on thoracic segments.

*Head* small (0.32 mm, ca. 8% of body length) subquadrate, clypeus arcuate, antennae high on head, at about the upper third, each an elongate oval with stronger lateral but weak anterior and posterior demarcation, the three weakly defined sensilla each with a small blunt subglobular spinule. Hairs on head very sparse (ca. 16–20) and short (approximately 5 μm): two on each side near the anterolateral margin of median portion of clypeus, two on each side near posterolateral margin of clypeus, one on the side of head behind the mandibular insertion, a short longitudinal row of 2–3 hairs on sides near posterolateral corners of head and a single hair on each side between these rows, behind the level of the antennae. Labrum subrectangular, slightly wider than long, with a row of four hairs on the anterior margin, a few rows of elongate spinules posteriorly on the ventral border and numerous rows on the posterior margin. Mandible pogonomyrmecoid, with a sharp-edged, strongly inwardly curved apical tooth and two very blunt preapical teeth. Maxillae paraboloidal, anterior and interior surfaces of the lacinia with rows of spinules, stipes without spinules, but with 3–4 hairs on the outer surface; the paxilliform maxillary palp stouter and sub-equal in length to the digitiform galea, both with apical and subapical sensilla. Anterior surface of labium with short rows of elongate spinules, labial palps paxilliform and ventrolaterally situated, with one subapical and three apical sensilla. Hypopharynx densely spinulose, the spinules arranged similarly to those on the posterior margin of the labrum.

Larval morphology is similar to that described for *Plectroctenacryptica* Bolton (Wheeler and Wheeler 1976b) and *P.mandibularis* Smith (listed as *P.conjugata* Santschi in Wheeler and Wheeler 1989). The number of tubercles in *B.ikhemka* (ca. 700) is intermediate between that reported for *P.mandibularis* (ca. 1600) and *P.cryptica* (ca. 350), the latter being similar to the 300 reported for *Streblognathusaethiopicus* (Smith) by Wheeler and Wheeler (1989). The spiracle structure in *Boloponera* most closely matches that described by Wheeler & Wheeler (1976a) for *Paraponera* and *Thaumatomyrmex*. Although the spiracle form in *Plectroctena* was not explicitly described for either *P.mandibularis* or *P.cryptica*, inspection of a *P.mandibularis* larva at 230× magnification suggests that the structure is similar in this genus; SEM examination would be required to confirm this.

## Discussion

### Related species and phylogenetic affinities

*Boloponeraikemkha* is readily distinguished from its only known congener by the highly polished torular lobes and lack of standing hairs on the scapes, mesosoma, petiole and first gastral tergite. The discovery of a second species in the genus has confirmed the consistency of several characteristics, as well as enabling the recognition of new autapomorphies, that are of significance in elucidating relationships with other closely related genera, as discussed below.

It is pertinent first to correct some errors in previous discussions of the relationship of *Boloponera* with other members of the *Plectroctena* genus-group. Firstly, Schmidt and Shattuck (2014) state that *Boloponera* has abundant short pilosity but no pubescence, but both *B.vicans* and *B.ikhemka* do in fact possess pubescence; *B.ikhemka*, however, almost entirely lacks standing pilosity. Secondly, Schmidt and Shattuck (2014), as well as Fisher (2006), described the propodeum of both *Boloponera* and *Plectroctena* as having posterolateral margins expanded into lamellae. However, reference to the descriptions in Bolton (1974), and inspection of images on AntWeb as well as specimens of three species represented in the AFRC, indicate that the majority of *Plectroctena* species have what can at most be described as a distinctly marginate propodeum (e.g. *P.strigosa*, Emery, illustrated here in Figure 3C, D). Some species have propodeal ridges that could be described as laminae but only a few could be considered to have lamellae (which are thin and generally translucent) developed to a significant degree. In contrast, both *B.vicans* and *B.ikhemka* have well-developed, thin and translucent lamellae that run the entire length of the margin between the lateral propodeum and the declivity. The presence of lamellae thus cannot be considered a synapomorphy linking *Plectroctena* with *Boloponera*. Thirdly, Schmidt and Shattuck (2014) state that, as in *Loboponera*, the anepisternum in *Plectroctena* appears fused to the mesonotum and metapleuron. While this may be the case for a few of the 16 *Plectroctena* species illustrated on AntWeb, in the majority of these, and in all three species represented in the AFRC, the anepisternum is moderately to well-defined by impressed sutures and is more distinct from the mesonotum and metapleuron than it is from the katepisternum. This character therefore cannot be considered a synapomorphy linking *Plectroctena* and *Loboponera*. In *B.vicans* and *B.ikemkha*, the anepisternum is contiguous with the metapleuron, but remains separated from the mesonotum by a weak but distinct suture.

Fisher (2006) noted similarities between *Boloponera* and *Plectroctena*, but also highlighted several differences and concluded that *Boloponera* should be excluded from the *Plectroctena* genus-group (*Loboponera* + *Plectroctena* + *Psalidomyrmex*) as defined by Bolton and Brown (2002). However, based on molecular and morphological evidence, Schmidt and Shattuck (2014) subsequently placed *Boloponera* within their expanded *Plectroctena* genus-group (*Boloponera* + *Centromyrmex* + *Dolioponera* + *Feroponera* + *Loboponera* + *Plectroctena* + *Psalidomyrmex*), as sister to *Plectroctena* and/or *Loboponera*, and suggested that *Boloponera* might even be nested within *Plectroctena*. The discovery of *B.ikhemka* has however confirmed several of the previously recognised differences between *Boloponera* and *Plectroctena* including:

1. the lack in *Boloponera* of a highly modified mandibular articulation (incorporating differences in the structure of the mandibular articulation itself as well as of the clypeus and genae);

2. the presence of both an apical and a preapical tooth on the mandible in *Boloponera*, as well as an additional tooth on the masticatory margin close to the basal tooth. *Plectroctena* have a blunt or truncated mandible with neither an apical nor a preapical tooth and, when present, the masticatory margin tooth is widely separated from the basal angle or tooth;

3. the absence in *Boloponera* of a longitudinal groove on the inner half of the dorsal mandibular surface running parallel to the masticatory margin;

4. the absence of a mesofemoral gland in *Boloponera*, and

5. the differing form of the anterior disc of the petiole in *Boloponera*.

Schmidt & Shattuck (2014: 162) downplayed the significance of the last character and, based on the rather indistinct published image of this feature in *B.vicans*, suggested that the shape of the median depression in the petiolar articulatory surface in *Boloponera* is more similar to that of *Plectroctena* than is its shape in *Loboponera*. However, this cannot be said of *B.ikhemka*, in which the median depression is very distinct from that of *Plectroctena* (compare Figure 2F with figures 28–29 of Fisher, 2006) and is more like a foreshortened version of that found in *Loboponera* (see figures 25, 26 in Fisher, 2006). The median depression in *Boloponera* is broadly rounded anteriorly and approximately equal in width to the horseshoe-shaped articulatory strip, which perfectly matches Bolton & Brown’s (2002) description of the ancestral structure. In strong contrast, in *Plectroctena* (and *Psalidomyrmex*), the median depression is reduced in width anteriorly to a very narrow groove by inward expansion of the articulatory surfaces (as described in Bolton & Brown, 2002), with an overall arrowhead-like appearance in most species. *Loboponera* appears intermediate, with the median depression significantly narrower than the lateral strips, but approximately uniform in width and by no means as compressed anteriorly as in *Plectroctena*. On current evidence I therefore disagree with Schmidt & Shattuck’s (2014) assessment of this character and consider *Boloponera* to display a more ancestral form, with *Loboponera* showing slight modification and *Plectroctena* + *Psalidomyrmex* with the most derived state.

*Boloponera*, *Loboponera* and *Plectroctena* share a longitudinal metafemoral gland as a synapomorphy, but *Boloponera* lacks the similar mesofemoral gland found in both *Loboponera* and *Plectroctena*. There is no visible groove in *Boloponera*, in which the gland is indicated merely by a thinning of the cuticle. However, the development of the grooves appears variable in the *Plectroctena* material I have inspected, and thus not much weight can be placed on this character; only the presence/absence of the mesofemoral glandular structure is of significance here and again *Boloponera* appears to display the less-derived state.

While Bolton and Brown (2002) considered the strongly arched A4 tergite autapomorphic in *Loboponera*, several *Plectroctena* (e.g. *P.dentata* Santschi, *P.strigosa* and *P.thaui* Fisher) have the A4 moderately arched (tergite approximately three times the length of the sternite) and in at least one (*P.laevior* Santschi), the A4 is even more strongly arched (tergite more than four times the length of the sternite) than that of some *Loboponera*, while most other *Plectroctena* species have the A4 slightly arched (tergite roughly twice the length of the sternite). The variability of this character within *Plectroctena* means that in addition to not being autapomorphic for *Loboponera*, it also cannot be considered a synapomorphy for *Plectroctena* + *Loboponera*. In *Boloponera* the A4 tergite (Figure 1B, C) is straight or weakly arched anteriorly and only weakly arched posteriorly, the tergite being approximately 2.5 times the length of the sternite. Although I believe that relatively little weight can be afforded to the character, *Boloponera* does again appear to have the least derived form, with *Plectroctena* and *Loboponera* showing increasing modification.

In contrast, the extreme protrusion of the torular lobes beyond the posterior clypeal margin, which itself is extended anterad of the anterior clypeal margin and overhanging the mouthparts, appears unique to *Boloponera* and hence more derived. While the anterior clypeal margin also overhangs the mouthparts in *Loboponera*, the torular lobes do not extend anterad of the clypeus as they do in *Boloponera*. In *Plectroctena* the torular lobes are less developed and the clypeus is approximately vertical, not overhanging the mouthparts. Movement of the antennal scapes in *Boloponera* (as well as *Loboponera*) is highly constrained by the enlarged torular lobes. Despite the complex shape of the basal portion of the scape (see Figure 2C), which allows some additional dorsal movement, the entire scape is prevented from rising above a plane at approximately the level of the top of the lobes. The adaptive significance of this is unknown.

The arrangement of clypeal setae described here for *B.ikemkha* appears identical in *B.vicans* and, at least within the *Plectroctena* genus-group, seems to be unique to the genus. Inspection of AntWeb images of all other Afrotropical members of the *Plectroctena* genus-group *sensu*Schmidt and Shattuck (2014), including 10 *Centromyrmex*, one *Dolioponera*, one *Feroponera*, nine *Loboponera*, 16 *Plectroctena* and six *Psalidomyrmex* species, did not reveal the pattern repeated. Although *Feroponeraferox* Bolton & Fisher, *Plectroctenaanops* Bolton, *Loboponeranobiliae* Fisher, *Centromyrmexbequaerti* (Forel), *C.praedator* Bolton & Fisher and *C.sellaris* Mayr each have a moderately convergent pair of clypeal setae, which in two cases meet at the tips, none have setae which cross substantially proximal of the tips as in *Boloponera* and in no case are there two divergent pairs in addition to the convergent pair. This also appears to be a character for which *Boloponera* displays the most modified state.

In both *Loboponera* and *Plectroctena*, as in all other members (except *Feroponera*) of Schmidt & Shattuck’s (2014) expanded *Plectroctena* genus-group, the metapleural gland opens laterally and is clearly visible in profile view, while both *Boloponera* species have the metapleural gland bulla strongly laterally expanded ventrally (visible in ventral view in Figure 2F and in dorsal view in Figure 3B), resulting in a dorsally oriented orifice (Figure 3B). This is obscured in profile view (Figure 3A), especially posteriorly, by the dorsad extension of the ventral flap on the metapleural gland opening; in this character *Boloponera* again shows an apparently more derived state than either *Loboponera* or *Plectroctena*.

The prora in *Boloponera* is fairly inconspicuous, while in *Plectroctena* the prora is evident as a much more prominent projection on the anterior face of the first gastral sternite; in *Loboponera* the prora is often exceptionally well developed and in several species forms a very prominent antero-ventral projection.

In summary, *Boloponera* and *Loboponera* are similar with respect to petiole articulation, the expansion of the torular lobes, orientation of the clypeal surface, mandibular articulation and absence of a dorsal groove on the mandible. *Loboponera* and *Plectroctena* are similar in having the torular lobes not overhanging the mouthparts, configuration of clypeal setae, presence of a mesofemoral gland, position of the metapleural gland opening, development of the prora and degree of arching of A4. *Plectroctena* and *Boloponera* are similar in the absence of posterolateral head flanges and shape of the mandibles, although the latter may not be equivalent in view of the extreme development of other mandibular characters in *Plectroctena*.

Thus there appear to be fewer characters suggesting a close relationship between *Boloponera* and *Plectroctena* than there are linking *Boloponera* with *Loboponera* or *Loboponera* with *Plectroctena*; this assessment implies that *Boloponera* and *Plectroctena* are less similar to each other than either is to *Loboponera*, suggesting an intermediate position for the latter.

The characters discussed above strongly support the retention of *Boloponera* as distinct from *Plectroctena* and suggest that *Boloponera* is sister to *Loboponera* and *Plectroctena* rather than being closer to or even nested within *Plectroctena*. Nesting of *Boloponera* within *Plectroctena* would imply either that, in addition to the appearance of several new unique characteristics in *Boloponera*, there had been secondary loss (with reversion to the ancestral state) of three *Plectroctena* autapomorphies relating to the mandibles and head capsule (mandible dentition, dorsal groove and articulation), or alternatively that *Plectroctena* is paraphyletic and that these same adaptations had arisen independently in two separate lineages. The latter is highly improbable, but even the former seems unlikely and the hypothesis that *Boloponera* split from an ancestral line, before the unique mandibular configuration of *Plectroctena* had arisen, is a far more parsimonious explanation.

Finally, were *Boloponera* to be included within *Plectroctena*, which ranges in total length from 7 mm to more than 18 mm, they would be by far the smallest representatives of the genus, both species being barely more than half the length of the smallest *Plectroctena* currently known, and hence resulting in a very disjunct size range. *Loboponera* includes species intermediate in size between *Boloponera* and *Plectroctena*, with lengths ranging from about 3–7 mm, which may provide further (if weak) support for *Loboponera* being intermediate between *Plectroctena* and *Boloponera*.

The question remains, which of the three genera is closest to the ancestral form and which is the most derived? The characters discussed here are equivocal, with each genus displaying some that appear most derived and others that appear least derived, suggesting that *Boloponera*, *Loboponera* and *Plectroctena* have all undergone substantial modification since separation of their respective lineages; more comprehensive DNA phylogenetic analysis will most likely be required to resolve this question.

After the above reassessment of characters linking and separating *Boloponera*, *Loboponera* and *Plectroctena*, only the presence of a longitudinal metafemoral gland remains as a synapomorphy linking all three genera, other synapomorphies each being shared by only two of the three genera.

### Ergatoid queens

Although there is no direct evidence that the *B.ikemkha* specimens with eyes are ergatoid queens, this seems the most likely explanation for the differences between the two distinct forms collected. In addition to having eyes, the larger specimens also have relatively larger gasters, with the width and length of gastral tergites 1 and 2 averaging 6–13% larger relative to the size of the mesosoma in specimens with eyes than in eyeless specimens. The relatively larger gasters would allow for development of ovaries. The lack of clear differences in the structures of the pronotum and mesonotum, where ergatoid queens normally exhibit distinguishing characters, may be due to the extreme fusion of segments in *Boloponera* (Christian Peeters, pers. comm.). Additional arguments in favour of the larger specimens being ergatoids rather than large workers are 1) it is very probable that *Boloponera* forage entirely underground, so there would be no apparent function for eyes in workers of any size, 2) it is also very likely that colony size in *Boloponera* is small and this would suggest that they could not afford the cost of producing size-variable workers (Christian Peeters, pers. comm.) and 3) analysis of morphological measurements indicates that there are discrete differences in relative proportions of body parts, including leg lengths (the specimens with eyes have relatively longer legs overall as well as differing relative leg lengths), which is different from what would be expected from a simple allometric relationship between leg length and body size within the worker caste. Ideally, dissection of colony samples or observation of live colonies in the laboratory should be carried out to confirm or refute the ergatoid hypothesis. However, given the extreme rarity of *Boloponera*, the opportunity to do this may not arise for a considerable time. One of the eyeless specimens has thus been designated as the holotype worker and the larger specimens with eyes treated as ergatoid queens, pending further investigation.

### Habitat and distribution

**Figure 5.**
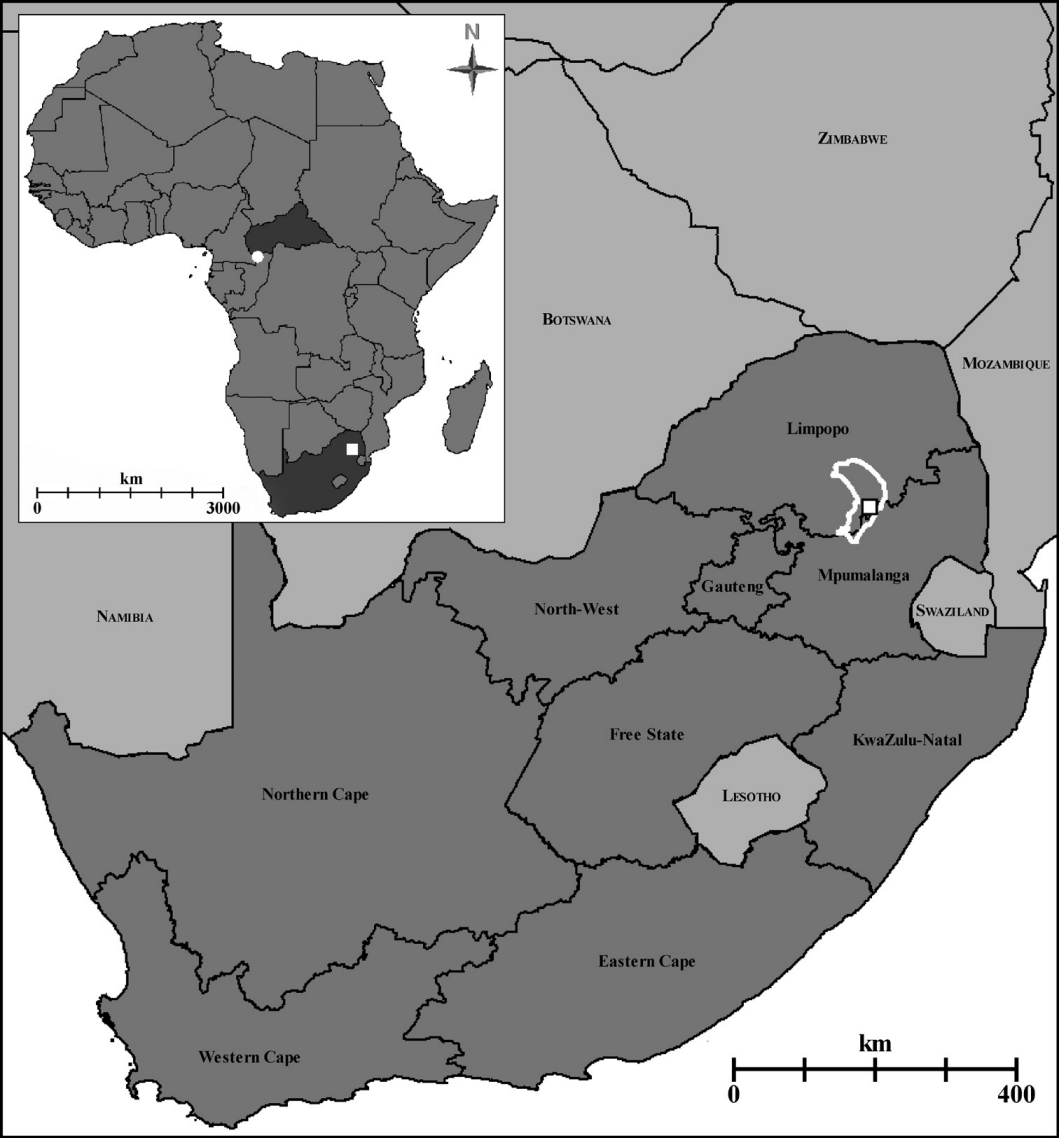
Map of Africa (inset, with Central African Republic and South Africa shaded dark grey) and detail of South Africa (main image), showing known distribution of *Boloponera*. Key: white circle - *B.vicans*; white square - *B.ikemkha*; white outline - Sekhukhuneland Centre of Plant Endemism.

*Boloponeraikemkha* was found within the Sekhukhuneland Centre of Plant Endemism (SCPE), an area recognised for its unique plant diversity (Siebert et al. 2002, 2010) but as yet very poorly known from an invertebrate perspective. The type locality of *B.ikemkha* is approximately 3400 km SSE of that of *B.vicans* (see Figure 5) and in a markedly different habitat with very different climatic conditions. While *B.vicans* was collected well within the Central African rainforest belt, *B.ikemkha* was found in a narrow strip of riparian woodland (canopy less than 10 m) in the much drier Sekhukhune Mountain Bushveld (*sensu*Mucina and Rutherford 2006). The mean annual rainfall is more than double in the *B.vicans* (1621 mm) than in the *B.ikemkha* (718 mm) type locality and seasonality of both precipitation (45.8 vs 79.6) and temperature (57.8 vs 387.9) also differ substantially (WorldClim dataset, Hijmans et al. 2005). While the subterranean conditions to which these ants are presumably mainly subjected are probably significantly buffered in comparison to the above-ground measured climate, it is remarkable that the two species inhabit such strongly contrasting habitats. Several other ant genera previously recorded in Africa only from tropical regions have recently been recorded in southern Africa; these include *Apomyrmastygia* Brown, Gotwald & Lévieux from the Kruger National Park, South Africa and *Aenictogiton* from northern Namibia and southern Angola (Parr et al 2003), as well as unpublished South African records in the AFRC of *Centromyrmexfugator* Bolton & Fisher from Gauteng and an undescribed *Anillomyrma* from Limpopo. In the case of *Apomyrma* and *Centromyrmex* the southern African material represents species that also occur in West and Central Africa respectively, but whether or not this is also the case cannot yet be determined for the *Aenictogiton* and *Anillomyrma* records. All of these genera are generally subterranean in their habits and thus, like *Boloponera*, are presumably buffered from climatic extremes when they occur outside their apparently normal range.

Further surveys may reveal the existence of additional *Boloponera* species and yield a less disjunct genus-level distribution, but unfortunately the currently available information does not provide any indication of the types of habitat that should be surveyed in order to maximise the chance of such discoveries being made. The *B.ikemkha* specimens were found during a brief *ad hoc* active sampling survey of riverine fringe vegetation within the predicted zone of influence of the proposed tailings storage facility at Two Rivers Platinum. No *Boloponera* specimens were located during far more intensive surveys, equivalent to the ALL-Protocol (Agosti et al. 2000), of three areas of other more open habitat types within the footprint of the proposed TSF, as well as at least 20 other ALL-Protocol equivalent surveys within 6 km and a further six within 11–27 km of this site, from 2007–2016. It therefore seems likely that *B.ikemkha* prefers the moister riverine fringe which has denser tree cover; such habitat forms only a small proportion of the total area of the SCPE.

The ultramafic soils (of igneous origin, high in magnesium and iron, with very low silica content) of the SCPE vary considerably in mineral composition and structure and this contributes to the complex pattern of very varied and unique plant communities, with many endemic species of limited distribution linked to particular soil forms (Siebert et al. 2001). Recent extensive sampling of ants in the region suggests that the same holds true for ant communities and species; for example two undescribed *Cardiocondyla* species are each apparently limited to particular soil types within the SCPE (unpublished data). It is possible that *B.ikemkha* is both rare and restricted to a particular habitat and soil type within the SCPE, but nothing is known about its ecology or behaviour.

### Conservation and threats

*Boloponera* is one of the most rarely encountered ant genera in Africa, with each of the two known species having been recorded only once to date. Whether this is an indication of true rarity, or an artefact of an extremely cryptic lifestyle, may be difficult to determine with certainty. However, *Hypoponera*, a related genus of cryptic, largely subterranean ants, with workers similar in size to or, more commonly, smaller than *Boloponera*, has 51 described species endemic to the Afrotropical region (Bolton and Fisher 2011). While some of these are also known from only a single collection, many are widespread and are frequently encountered in soil at depths similar to that at which *B.ikemkha* was found. During a survey of leaf litter ants in Ghana, Belshaw and Bolton (1994) collected 2410 specimens of *Hypoponera* representing six species, with a range from six to 1828 individuals per species. Unless *Boloponera* are very significantly more cryptic and deeply subterranean, this suggests that they are far rarer than *Hypoponera*, probably by at least 2–3 orders of magnitude.

The type locality of *B.ikemkha*, ca. 5 km east of the confluence of the Groot and Klein Dwars Rivers, lies approximately 400 m from the nearest boundary of the proposed new tailings storage facility for TRPM. Very little similar habitat lies within the proposed TSF site. However, approximately 2 km of this riverine fringe vegetation lies within 350–900 m down-slope of the proposed TSF. A further 1 km (within which the type locality lies) falls within 350–700 m of the proposed TSF but is separated from it by a low (10–30 m high) ridge. Dust clouds emanating from existing tailings dams in the region are frequently observed rising well over 500 m into the air and dispersing over distances of at least 8 km. It is thus clear that significant dust pollution can be expected close to the proposed new TSF unless far more effective dust control is implemented in the new facility. In addition, while possible seepage of contaminated water could not directly affect the *B.ikemkha* type locality due to the intervening ridge, such pollution could impact on the 2 km section of likely habitat down-slope of the proposed TSF, which thus poses a potential threat to the survival at least of part of the local population of the species; it is thus imperative that effective water management and dust control measures be implemented.

The Groot and Klein Dwarsrivier valleys and surrounding areas are subject to intense mining and prospecting pressure, with at least eight operational mines within an eight km radius of the *B.ikemkha* type locality and more mines further afield. Many more mines are planned for this region, which forms part of the Bushveld Igneous Complex, recognised to contain the richest deposits of platinum-group elements (PGE) in the world, representing 80% of known PGE reserves (Cawthorn 2010). There is also considerable habitat transformation due to increasing human populations (in part caused by an influx of job-seekers attracted by the mines) with concomitant increases in subsistence agriculture and livestock grazing. The cumulative effect of anthropogenic impacts and mining operations on natural habitats in the region may become highly significant and could pose a serious threat to the survival of *B.ikemkha*, as well as that of other species endemic to Sekhukhuneland. Habitat transformation and fragmentation would be especially significant for *B.ikemkha* if the species does, as seems most likely, have ergatoid queens and hence very limited dispersal abilities.

Application of the IUCN Red List Criteria (IUCN 2012) to the currently available data would indicate that *B.ikemkha* should be classified as Critically Endangered (CR B1ab(iii)+2ab(iii)), since it has been recorded from only a single locality which is currently under threat and other potential habitat in the region is also under threat. However, the species could alternatively be considered as Data Deficient and in urgent need of investigation. Additional surveys could either confirm a CR status or result in a lower threatened category such as Endangered (EN) or Vulnerable (VU) being assigned, but it is highly unlikely that a category lower than VU will result. Formal Red List assessments of the conservation status of species such as *B.ikemkha* may enable action to be taken to protect sufficient areas of natural habitat to ensure their continued survival and such assessments should be undertaken as a matter of urgency.

## Conclusions

The discovery of a second species of *Boloponera* confirms the consistency of several previously recognised as well as some newly identified autapomorphies for the genus and provides support for its retention as distinct from *Plectroctena*, although full relationships between *Boloponera*, *Loboponera* and *Plectroctena* remain unresolved. *Boloponeraikemkha* is considered to be extremely rare and is believed to be threatened by current and planned mining activities in the Sekhukhuneland Centre of Plant Endemism.

## Supplementary Material

XML Treatment for Boloponera ikemkha
